# RPC-Based Orthorectification for Satellite Images Using FPGA

**DOI:** 10.3390/s18082511

**Published:** 2018-08-01

**Authors:** Rongting Zhang, Guoqing Zhou, Guangyun Zhang, Xiang Zhou, Jingjin Huang

**Affiliations:** 1 School of Precision Instrument and Opto-Electronic Engineering, Tianjin University, Tianjin 300072, China; zrt65@tju.edu.cn (R.Z.); jingjin_huang@tju.edu.cn (J.H.); 2 Guangxi Key Laboratory for Spatial Information and Geomatics, Guilin University of Technology, Guilin 541004, China; zqx0711@tju.edu.cn; 3 The Center for Remote Sensing, Tianjin University, Tianjin 300072, China; guangyunzhang1@gmail.com; 4 School of Microelectronics, Tianjin University, Tianjin 300072, China

**Keywords:** orthorectification, field-programmable gate array (FPGA), rational polynomial coefficient (RPC)

## Abstract

Conventional rational polynomial coefficients (RPC)-based orthorectification methods are unable to satisfy the demands of timely responses to terrorist attacks and disaster rescue. To accelerate the orthorectification processing speed, we propose an on-board orthorectification method, i.e., a field-programmable gate array (FPGA)-based fixed-point (FP)-RPC orthorectification method. The proposed RPC algorithm is first modified using fixed-point arithmetic. Then, the FP-RPC algorithm is implemented using an FPGA chip. The proposed method is divided into three main modules: a reading parameters module, a coordinate transformation module, and an interpolation module. Two datasets are applied to validate the processing speed and accuracy that are achievable. Compared to the RPC method implemented using Matlab on a personal computer, the throughputs from the proposed method and the Matlab-based RPC method are 675.67 Mpixels/s and 61,070.24 pixels/s, respectively. This means that the proposed method is approximately 11,000 times faster than the Matlab-based RPC method to process the same satellite images. Moreover, the root-mean-square errors (RMSEs) of the row coordinate (Δ*I*), column coordinate (Δ*J*), and the distance Δ*S* are 0.35 pixels, 0.30 pixels, and 0.46 pixels, respectively, for the first study area; and, for the second study area, they are 0.27 pixels, 0.36 pixels, and 0.44 pixels, respectively, which satisfies the correction accuracy requirements in practice.

## 1. Introduction

Orthorectification is a process that orthorectifies an image onto its upright planimetry map and removes the perspective angle [1,2,3]. Orthorectification is a prerequisite for remotely sensed (RS) image applications in areas such as land resource investigation, disaster monitoring, forestry inventory, and environmental changes analysis. The RS image that is orthorectified not only contains the geometric accuracy of the map but also has the features of the remote sensing image. In the past 20 years, many orthorectification methods were proposed. For example, Zhou et al. [2] presented a comprehensive study on theories, algorithms, and methods of large-scale urban orthoimage generation. Zhou [3] proposed a near real-time orthorectification method for mosaic of video flow acquired by an unmanned aerial vehicle (UAV). Aguilar et al. [4] used rigorous model and rational function model to orthorectify GeoEye-1 and WorldView-2 images and assessed the geometric accuracy of the orthophoto. The results showed that the best horizontal geo-positioning accuracies were acquired by using third order rational functions with vendor’s RPC coefficients data. Marsetič et al. [5] presented an automatic processing chain for orthorectification of optical pushbroom sensors. Habib et al. [6] proposed an approach using generated orthophotos from frame camera to improve the orthorectification of hyperspectral pushbroom scanner imagery. However, these studies on orthorectification were based almost entirely on ground-image processing systems, which is unable to meet the demand with respect to time-critical disasters. Thus, it is important to determine how to improve the speed of the orthorectification process when used in the on-board processing of a spacecraft.

With the increasing demands in (near) real-time RS imagery applications for applications such as military deployments, quick response to terrorist attacks, and disaster rescue (e.g., flooding monitoring), the on-board implementation of orthorectification has attracted much research worldwide in recent years. To increase the speed of image processing, researchers have proposed multiple parallel-processing methods and employed hardware acceleration such as the approach by Warpenburg and Siegel [7], who performed resampling in a single instruction stream-multiple data stream environment. Wittenbrink et al. [8] presented optimal concurrent-read-exclusive-write and exclusive-read-exclusive-write parallel-random-access-machine algorithms for spatial image warping. Liu et al. [9] proposed a parallel algorithm that is focused on massive remotely sensed orthorectification. Dai and Yang [10] proposed a fast graphic processing unit (GPU)–central processing unit (CPU) cooperative processing algorithm that is based on computing unified device architecture for the orthorectification of RS images. Reguera-Salgado et al. [11] proposed a method for the real-time geocorrection of images from airborne pushbroom sensors using the hardware acceleration and parallel-computing characteristics of modern GPUs. Quan et al. [12] presented an optical aerial image orthorectification parallel algorithm that employs GPU acceleration. These ground-based parallel-processing systems have increased to an extent the processing speed for RS image orthorectification. However, the RS images still need to be sent back to the ground-based processing centers. However, this process is time consuming. In addition, most parallel-processing methods are based on the multiple task operating system of the GPU, which cannot essentially solve the problem of a serial instruction method.

To realize on-board orthorectification in (near) real-time, an efficient approach is to apply field-programmable gate array (FPGA) hardware architecture because FPGA chips offer a highly flexible design, scalable circuits, and a high efficiency in data processing for its pipeline structure and fine-grained parallelism. In recent decades, researchers have widely used FPGA for image processing applications. Examples are Halle can coworkers’ [13] proposed on-board image data processing system based on the neural network processor NI100, digital signal processors, and FPGA. Eadie et al. [14] investigated the use of FPGA for the correction of geometric image distortion. Kumar et al. [15] realized the real-time correction of images using an FPGA under a dynamic environment. Escamilla-Hernández et al. [16] and Kate [17] used an FPGA to implement data compression. Tomasi et al. [18] proposed a stereo vision algorithm using an FPGA to perform the correction of video graphics array images (57 fps). Pal et al. [19], Wang et al. [20], and Zhang et al. [21] applied FPGAs to accelerate the image data and signal filtering processes. Ontiveros-Robles et al. [22,23] proposed FPGA-based hardware architectures for real-time edge detection using fuzzy logic algorithm. Li et al. [24,25] utilized FPGAs to realize the real-time processing of video images to remove snow and fog. Huang et al. [26] proposed an FPGA-based method for the on-board detection and matching of the feature points. Huang et al. [27] presented a new FPGA architecture of a fast and brief algorithm for on-board corner detection and matching.

To the best of our understanding, research into FPGA hardware systems has focused mainly on the real-time correction of video images, noise removal, edge detection, etc., and there are few studies related to on-board orthorectification. Zhou et al. [28] first presented the concept of “on-board geometric correction”, but details pertaining to its on-board implementation were not given. Zhou [3] proposed a method for a real-time mosaic of video flow acquired by a small low-cost unmanned aerial vehicle. However, the method was implemented based on software, a serial instruction system, which would affect the real-time processing efficiency. Thus, this paper proposes a FPGA-based method for the on-board implementation of orthorectification. The proposed method can be divided into three modules: reading parameters module, coordinates transformation module, and interpolation module.

The major contribution of this study is a FPGA-based method, in which a traditional orthorectification algorithm is modified for on-board image (near) real-time orthorectification.

The paper is organized as follows. Section 2 describes the proposed RPC algorithm, i.e., fixed-point-based RPC (FP-RPC) algorithm, and gives the FPGA implementation process of the FP-RPC algorithm. Section 3 provides an experimental comparison of the proposed method using IKONOS-2 data and SPOT-6 data. Section 4 discusses the rectification accuracy by FPGA and PC platforms, and processing speed and resource consumption of these two platforms. Finally, Section 5 gives some conclusions.

## 2. RPC-Based Orthorectification Using an FPGA Chip

High-resolution satellite sensors are different from conventional aerial frame perspective imaging, and generally apply linear-array CCD pushbroom imaging technology. To deal with various types of images, many geometric processing models and algorithms are presented. One of the most widely used models is the rational polynomial coefficient (RPC) model, which is a general imaging model that is independent of the satellite sensor and platform. Many modern satellite images are equipped with rational polynomial coefficients (RPCs). Unlike rigorous physical models that are based on the collinear equation, which uses the ephemeris, attitude information, etc., to establish the acquisition geometry of the sensors, the RPC model does not require knowledge of the interior orientation elements and exterior orientation elements, which are sometimes not provided by vendors. The RPC model can produce uniform accuracy with a rigorous physical model, and is a simple generalized model. The RPC model has been widely applied to orthorectify satellite images with the increasing utilization of high-resolution images, as in [29,30,31,32,33,34,35]. The details of RPC orthorectification are given in [29,30].

In this study, the RPC algorithm is implemented using FPGA. Usually, an FPGA chip can offer a highly flexible design, scalable circuits, and a high efficiency in data processing for its pipeline structure and fine-grained parallelism. Moreover, an FPGA chip has advantages in size, weight, and power (SWaP) compared to GPU and CPU, which is helpful to integrate the FPGA into the on-board system.

However, the traditional RPC algorithm for orthorectification is computationally costly because of floating-point operations in the RPC algorithm. To implement on-board orthorectification using an FPGA chip in (near) real-time, a fixed-point-based RPC (FP-RPC) algorithm is proposed that can reduce the computation cost significantly. The details of the proposed method are given below.

### 2.1. Proposed RPC Algorithm

FP processing is a method that accelerates the calculation [36,37]. To make the transformation between a fixed-point variable and a floating-point variable, multiplication by a constant is necessary to maintain the precision. When the constant is set to a power of 2, the multiplication can be seen as a single bit shift, i.e.,

(1) $$F=\left\lfloor 2^{\tau }F^{\prime }\right\rfloor$$

where *F*′ is a floating-point variable, *F* is a fixed-point variable, and *τ* is a scale factor, which affects the binary accuracy of the resulting integer representation. A larger scale factor will produce a higher degree of the binary accuracy.

In the proposed FP-RPC algorithm, all of the variables and constants are transformed to integers using Equation (1). Table 1 gives the integer variables and their scale factors. In Table 1, *a*′*_i_*, *b*′*_i_*, *c*′*_i_*, and *d*′*_i_* (*i* = 1 to 20) are multinomial coefficients. Generally, the values of *b*′_1_ and *d*′_1_ are 1. *Lon*′, *Lat*′, and *Hei*′ are geodetic coordinates, which represent the longitude, latitude, and height, respectively. *Lat*′*_off_*, *Lat*′*_scale_*, *Lon*′*_off_*, *Lon*′*_scale_*, *H*′*_off_*, *H*′*_scale_*, *Line*′*_off_*, *Line*′*_scale_*, *Samp*′*_off_*, and *Samp*′*_scale_* are the parameters for normalization. *Samp*′ and *Line*′ represent the image coordinates, sample and line.

According to Fraser et al. [29] and Grodecki et al. [30] and Equation (1), the normalized coordinates are converted into integers by

(2) $$L=2^{\tau_{2}}\frac{2^{-\tau_{2}}Lon-2^{-\tau_{3}}Lon_{off}}{2^{-\tau_{3}}Lon_{scale}}\;P=2^{\tau_{2}}\frac{2^{-\tau_{2}}Lat-2^{-\tau_{3}}Lat_{off}}{2^{-\tau_{3}}Lat_{scale}}\;H=2^{\tau_{2}}\frac{2^{-\tau_{2}}Hei-2^{-\tau_{3}}H_{off}}{2^{-\tau_{3}}H_{scale}}$$

(3) $$X=2^{\tau_{2}}\frac{2^{-\tau_{2}}Samp-2^{-\tau_{3}}Samp_{off}}{2^{-\tau_{3}}Samp_{scale}}\;Y=2^{\tau_{2}}\frac{2^{-\tau_{2}}Line-2^{-\tau_{3}}Line_{off}}{2^{-\tau_{3}}Line_{scale}}$$

Moreover, the polynomials are converted into integers by

(4) $$2^{-\tau_{2}}ND_{\mathrm{LS}}=(2^{-\tau_{1}}C)(2^{-\tau_{2}}N^{T})$$

(5) $${\mathrm{ND}}_{\mathrm{LS}}=2^{-\tau_{1}}{\mathrm{CN}}^{T}$$

where

$${\mathrm{ND}}_{\mathrm{LS}}={\left[\begin{array}{cccc} Num_{L} & Den_{L} & Num_{S} & Den_{S} \end{array}\right]}^{T},$$

$$C=\left[\begin{array}{ccccc} a_{1} & a_{2} & \ldots & a_{19} & a_{20}\\ b_{1} & b_{2} & \ldots & b_{19} & b_{20}\\ c_{1} & c_{2} & \ldots & c_{19} & c_{20}\\ d_{1} & d_{2} & \ldots & d_{19} & d_{20} \end{array}\right],$$

$$N=\left[\begin{array}{cccccccccccccccccccc} 1 & L & P & H & LP & LH & PH & LL & PP & HH & PLH & LLL & LPP & LHH & LLP & PPP & PHH & LLH & PPH & HHH \end{array}\right].$$

In addition, the normalized image coordinates (*X*′, *Y*′) are converted into integers by 

(6) $$2^{-\tau_{2}}Y=\frac{2^{-\tau_{2}}Num_{L}}{2^{-\tau_{2}}Den_{L}},\;2^{-\tau_{2}}X=\frac{2^{-\tau_{2}}Num_{S}}{2^{-\tau_{2}}De{n^{\prime }}_{S}}$$

(7) $$Y=2^{\tau_{2}}\frac{Num_{L}}{Den_{L}},\;X=2^{\tau_{2}}\frac{Num_{S}}{Den_{S}}$$

Finally, the image coordinates (*Samp*′, *Line*′) are converted into integers by

(8) $$\left\{ \begin{array}{l} 2^{-\tau_{3}}Samp=(2^{-\tau_{2}}X)(2^{-\tau_{3}}Samp_{scale})+2^{-\tau_{3}}Samp_{off}\\ 2^{-\tau_{3}}Line=(2^{-\tau_{2}}Y)(2^{-\tau_{3}}Line_{scale})+2^{-\tau_{3}}Line_{off} \end{array}\right.\;$$

(9) $$\left\{ \begin{array}{l} Samp=(2^{-\tau_{2}}X)Samp_{scale}+Samp_{off}\\ Line=(2^{-\tau_{2}}Y)Line_{scale}+Line_{off} \end{array}\right.$$

### 2.2. Parallel Computation of Orthorectification Using an FPGA

**Figure 1:**
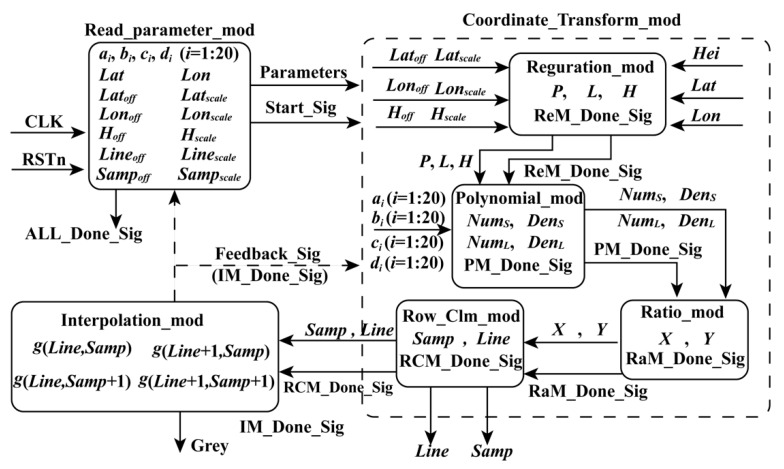
Flowchart showing implementation on an FPGA chip.

Many factors affect the computation speed when an FPGA is adopted, such as the optimal design of algorithms and the logical resource of the utilized FPGA. By analyzing the structure of the FP-RPC algorithm and optimizing it, an FPGA-based hardware architecture for FP-RPC-based orthorectification is designed, as shown in Figure 1. As described in Equations (2)–(9), their structures are similar. It is convenient for FPGAs to be implemented in parallel. As shown in Figure 1, the FPGA-based FP-RPC module can be divided into three submodules, that is, Read_parameter_mod (RPM), which is used to send parameters to other modules; Coordinate_Transform_mod (CTM), which is applied to transform geodetic coordinates to image coordinates; and Interpolation_mod (IM), which is utilized to perform bilinear interpolation. The details of these modules are given as follows.

1. For RPM, the coefficients of RPC can be calculated by least-squares adjustment [38]. According to Tao et al. [38], the computing processes of the RPC coefficients are as follows. Equation (7) can be rewritten as
  (10) $$F_{X}=Num_{S}(P,L,H)-2^{\tau_{2}}XDen_{S}(P,L,H)=0$$
  (11) $$F_{Y}=Num_{L}(P,L,H)-2^{\tau_{2}}YDen_{L}(P,L,H)=0$$
  Thus, the matrix form of error equation can be expressed as
  (12) V=MA−R
  where
  $$M=\left[\begin{array}{cccc} \frac{\partial F_{X}}{\partial a_{i}} & \frac{\partial F_{X}}{\partial b_{j}} & \frac{\partial F_{X}}{\partial c_{i}} & \frac{\partial F_{X}}{\partial d_{j}}\\ \frac{\partial F_{Y}}{\partial a_{i}} & \frac{\partial F_{Y}}{\partial b_{j}} & \frac{\partial F_{Y}}{\partial c_{i}} & \frac{\partial F_{Y}}{\partial d_{j}} \end{array}\right]\;(i=1,\;2,\;\ldots ,\;20;\;j=2,\;\ldots ,\;20)$$
  $$R={\left[\begin{array}{cc} -F_{X}^{0} & -F_{Y}^{0} \end{array}\right]}^{T}$$
  $$A={\left[\begin{array}{cccc} a_{i} & b_{j} & c_{i} & d_{j} \end{array}\right]}^{T}$$
  Equation (12) is solved by least-squares algorithm, and the solutions of RPC coefficients can be represented as
  (13) $$A={\left(M^{T}M\right)}^{-1}M^{T}R$$
  The solution for Equation (13) is acquired by an iterative process. The entire algorithm of Equation (13) has been implemented in our previous work [39], in which the detailed implementing process can be found.
  The normalization parameters can be calculated by the following equations.
  (14) $$Lat_{off}=\frac{\sum_{i=1}^{n}Lat_{i}}{n},\;Lon_{off}=\frac{\sum_{i=1}^{n}Lon_{i}}{n},\;H_{off}=\frac{\sum_{i=1}^{n}Hei_{i}}{n},\;Samp_{off}=\frac{\sum_{i=1}^{n}Samp_{i}}{n},\;\;Line_{off}=\frac{\sum_{i=1}^{n}Line_{i}}{n}$$
  (15) $$Lat_{scale}=\max\left(\left|Lat_{\max}-Lat_{off}\right|,\left|Lat_{\min}-Lat_{off}\right|\right),\;\;Lon_{scale}=\max\left(\left|Lon_{\max}-Lon_{off}\right|,\left|Lon_{\min}-Lon_{off}\right|\right)$$
  (16) $$H_{scale}=\max\left(\left|Hei_{\max}-H_{off}\right|,\left|Hei_{\min}-H_{off}\right|\right),\;\;Line_{scale}=\max\left(\left|Line_{\max}-Line_{off}\right|,\left|Line_{\min}-Line_{off}\right|\right)$$
  (17) $$Samp_{scale}=\max\left(\left|Samp_{\max}-Samp_{off}\right|,\left|Samp_{\min}-Samp_{off}\right|\right)$$
  where *n* is the number of ground control points (GCPs).
  When the enable signal is being received, the geodetic coordinates (*Lon*, *Lat*, *Hei*) stored in the RAMs are read, and sent to Coordinate_Transform_mod (CTM) with the attained parameters and the start signal (Start_Sig) in the same clock cycle.
2. When the Start_Sig, the constants, and the geodetic coordinates are being received in the CTM, the normalized coordinates (*P*, *L*, *H*) are first calculated in the regularization module (Regulation_mod, ReM). Then, the normalized coordinates and the done signal of ReM (ReM_Done_Sig) are sent to the polynomial module (Polynomial_mod, PM) with *a_i_*, *b_i_*, *c_i_*, and *d_i_* (*i* = 1 to 20) in the same clock cycle to compute the numerators and denominators (*Num_L_*, *Num_S_*, *Den_L_*, and *Den_S_*) of Equation (7). Subsequently, when the done signal (PM_Done_Sig) of PM, *Num_L_*, *Num_S_*, *Den_L_*, and *Den_S_* are being received, the normalized coordinates (*X*, *Y*) of the image coordinates are calculated. Finally, when the normalized coordinates (*X*, *Y*) and the done signal (RaM_Done_Sig) of the ratio module (Ratio_mod, RaM) are being received, the image coordinates (*Samp*, *Line*) and the done signal (RCM_Done_Sig) of the image coordinate calculation module (Row_Clm_mod, RCM) are acquired and sent to the interpolation module (Interpolation_mod, IM) in the same clock cycle.
3. When the image coordinates (*Samp*, *Line*) and RaM_Done_Sig are being received in IM, the gray of pixel (*Samp*, *Line*) is obtained by interpolating, and the done signal (IM_Done_Sig) of IM is produced.
4. When the posedge clk of the signal, ALL_Done_Sig is being detected, the processing is finished.

#### 2.2.1. Read Parameter Module

**Figure 2:**
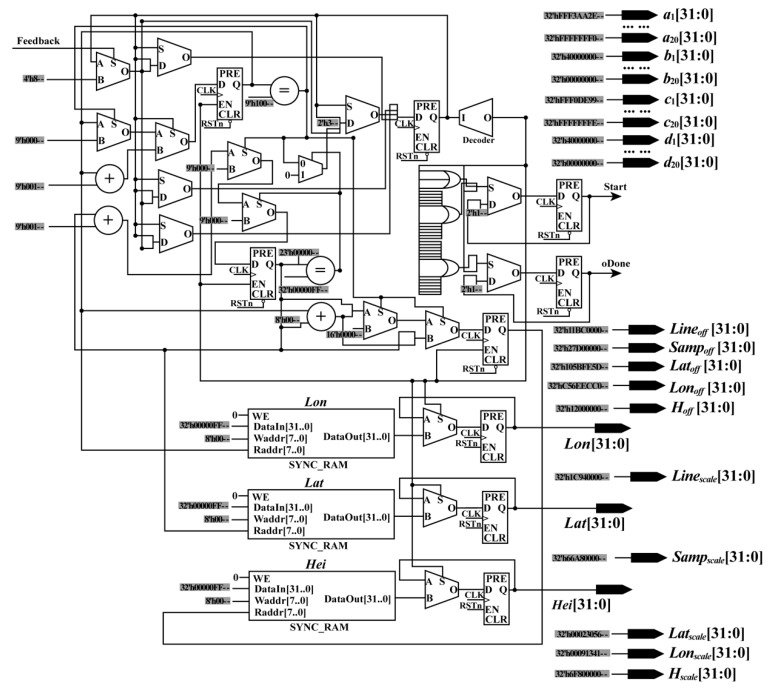
Schematic diagram of the read parameter module.

To ensure that the constants, geodetic coordinates, and the start signal (Start_Sig) are sent in the same clock cycle, a parallel module (i.e., the RPM) is designed (see Figure 2). In the RPM, the constants are assigned corresponding values, while the geodetic coordinates are stored in RAM. In this design, all values are expressed using a fixed point of 32 bits to ensure computational accuracy.

In the RPM, the geodetic coordinates are sent to the next module according to the order of the column. First, the address of RAM is initialized as 0. When the enable signal is detected, the first group of geodetic coordinates (*Lat*_0_, *Lon*_0_, *Hei*_0_) is read from the RAM and sent to the next module with the constants and the Start_Sig in the same clock cycle. Starting from the second group of geodetic coordinates, the rules for reading and sending geodetic coordinates are changed. In other words, after the second group of geodetic coordinates (*Lat*_1_, *Lon*_1_, *Hei*_1_), the geodetic coordinates will be read and sent unless the enable signal and the feedback signal (Feedback_Sig), which are sent by the interpolation module, are detected at the same time. After the final group of geodetic coordinates are read and sent, if the Feedback_Sig is received, the done signal (ALL_Done_Sig) of orthorectification is produced. When the ALL_Done_Sig is detected, the process of orthorectification is stopped.

#### 2.2.2. Coordinate Transformation Module

As shown in Figure 1, for the CTM, the inputs contain the constants, the geodetic coordinates, and the Start_Sig, while the outputs include image coordinates and the done signal of this module. The CTM can be divided into four submodules, namely ReM, PM, RaM, and RCM. Details regarding these four submodules are as follows.

**• Regulation Module**

**Figure 3:**
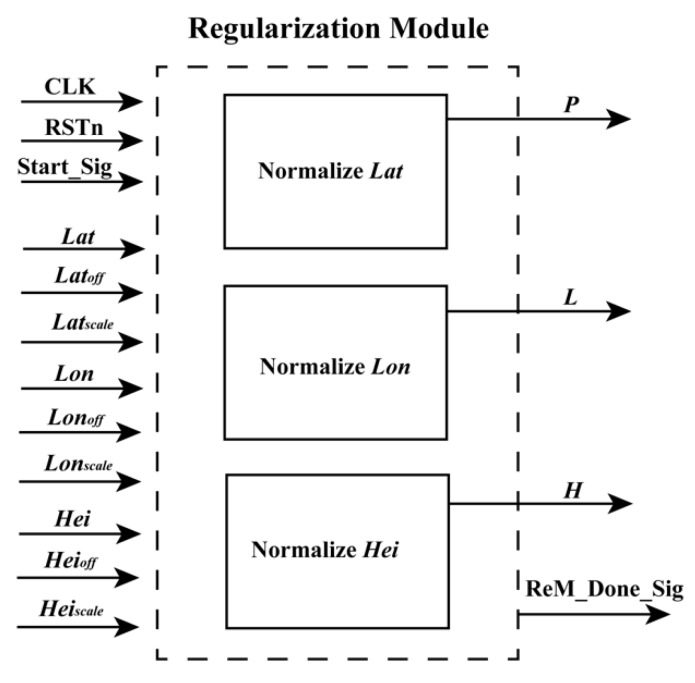
Schematic diagram of the ReM.

**Figure 4:**
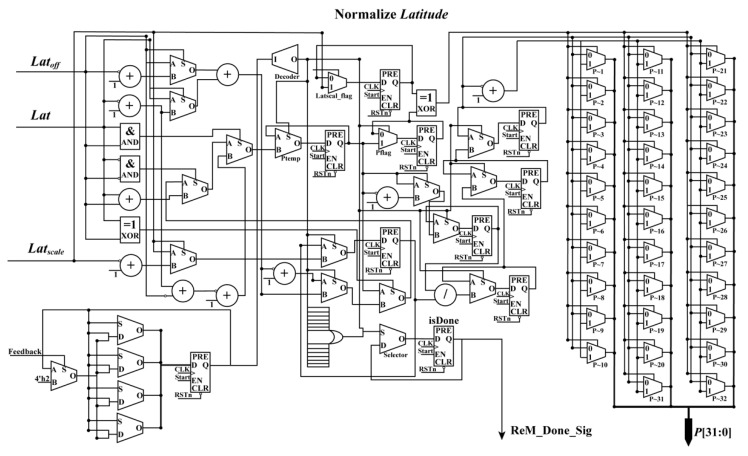
Schematic diagram of normalizing *Latitude*.

According to Section 2.1, the geodetic coordinates (*Lat*, *Lon*, *Hei*) should be first transformed as the normalized coordinates (*L*, *P*, *H*) based on Equation (2) because this operation can minimize the introduction of errors during the computation of the numerical stability of equations [13]. As shown in Equation (2), the forms of these equations are uniform. In other words, they are suitable for implementation using FPGA. To obtain the normalized coordinates (*L*, *P*, *H*) of the geodetic coordinates (*Lat*, *Lon*, *Hei*) using an FPGA chip, a parallel computation architecture is presented in Figure 3. In Figure 3, the structures of “Normalize *Lat*”, “Normalize *Lon*”, and “Normalize *Hei*” are similar. Thus, only the schematic diagram of “Normalize *Lat*” is presented (see Figure 4).

As shown in Figure 4, during the computation process, 1 divider, 10 adders, 10 flipflops, and 16 multiplexer units are mainly used to normalize the *Lat*. In this design, the relationship among “Normalize *Lat*”, “Normalize *Lon*”, and “Normalize *Hei*” is parallel. The normalized coordinates (*L*, *P*, *H*) are obtained in the same clock cycle as the done signal.

**• Polynomial Module**

**Figure 5:**
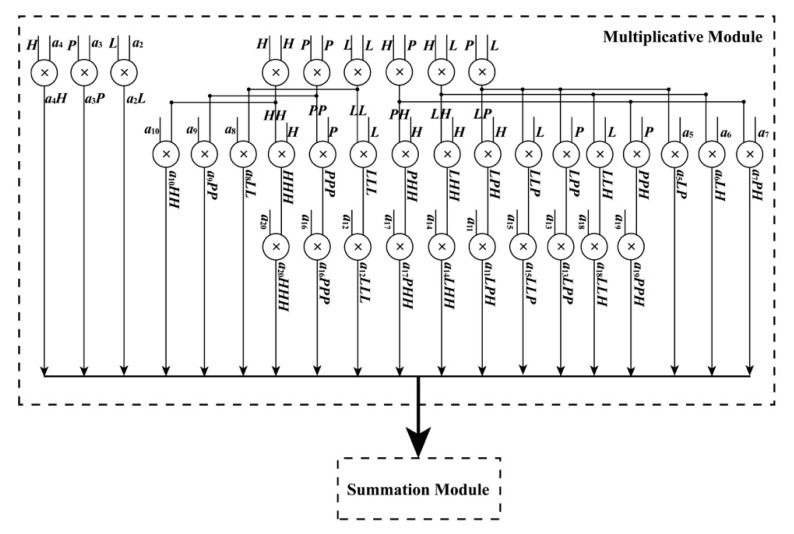
Schematic diagram of polynomial module.

**Figure 6:**
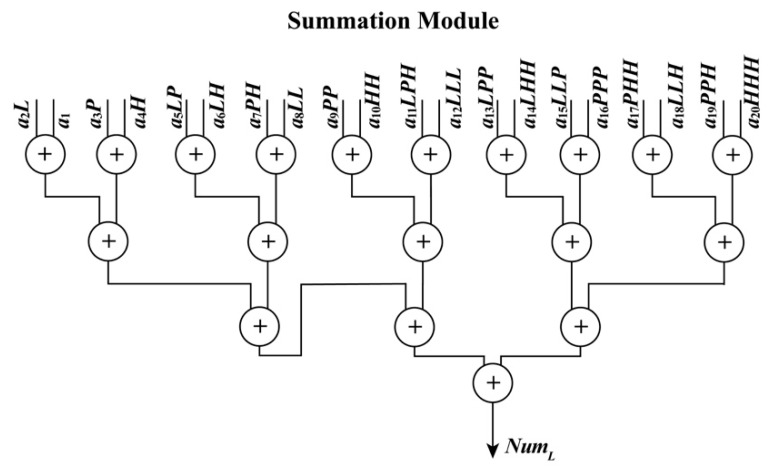
Schematic diagram of summation module.

**Figure 7:**
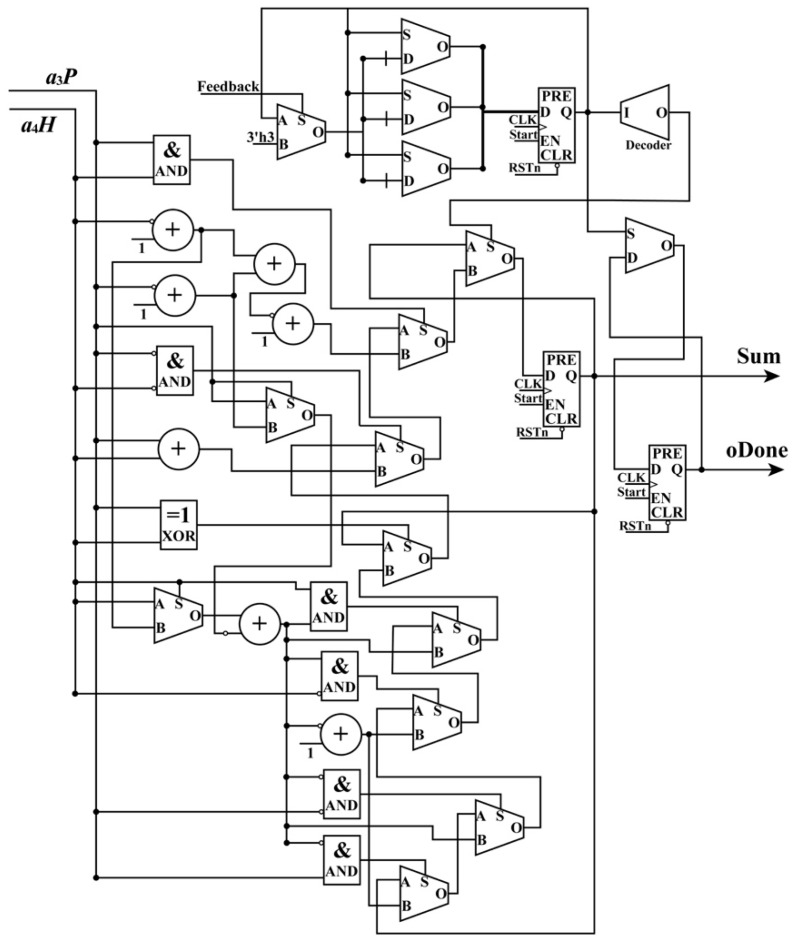
Details showing an example of extended addition.

When the ReM_Done_Sig and (*L*, *P*, *H*) are being received by the PM module, the PM module starts to work. As shown in Equations (4) and (5), these polynomials have a uniform form, which are suitable for the implementation of an FPGA chip in parallel. In these equations, variables such as *LH*, *LP*, and *PH* are shared. To implement these polynomials in parallel using an FPGA chip, a parallel computation architecture is proposed in Figure 5 and Figure 6. As shown in those figures, the PM module is divided into two parts: one is used to perform multiplication and the other is applied to manipulate addition. When performing addition, some special operations about the positive and negative sets of data should be considered. Thus, for the additions in Figure 6, each of them is extended to a similar form, as shown in Figure 7, taking the addition between *a*_3_*P* and *a*_4_*H* as an example. In the example, three situations are considered: (i) *a*_3_*P* and *a*_4_*H* are both positive; (ii) *a*_3_*P* and *a*_4_*H* are both negative; and (iii) *a*_3_*P* and *a*_4_*H* have opposite signs. The details for an extended addition are shown in Figure 7.

To implement each polynomial, 35 multipliers are utilized in the multiplication, and 19 extended additions are used. In each extended addition, three flipflops, four selectors, seven adders, and eleven multiplexers are applied. After processing the PM module, four sums, i.e., *Num_L_*, *Num_S_*, *Den_L_*, and *Den_S_*, are obtained with the done signal of the PM module, PM_Done_Sig, in the same clock cycle.

**• Ratio Module**

**Figure 8:**
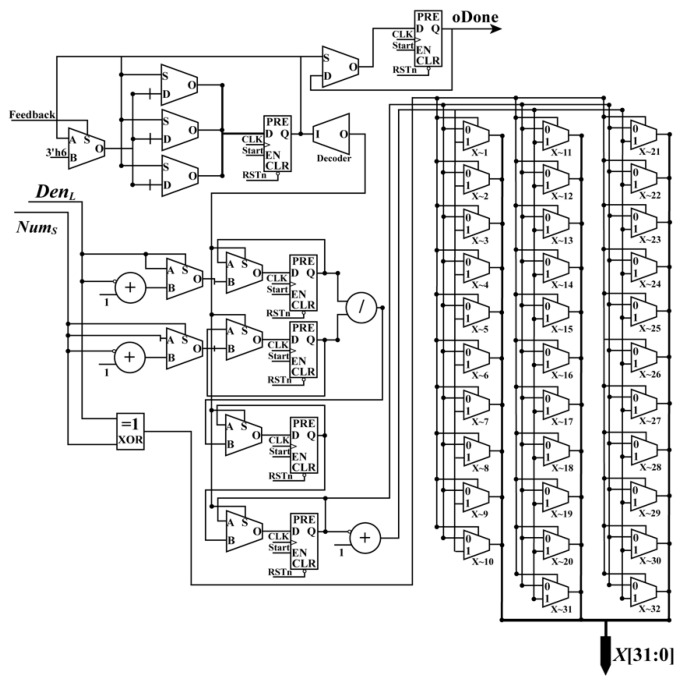
Schematic diagram of the ratio module.

When the PM_Done_Sig, *Num_L_*, *Num_S_*, *Den_L_* and *Den_S_* are being received, the RaM module starts to calculate the normalized coordinates (*X*, *Y*) of image coordinates. As shown in Equation (6), the forms for the two equations are the same. It is convenient to calculate *X* and *Y* in parallel using an FPGA chip. In Figure 8, a parallel-computing architecture that is used to calculate *X* is presented. In the same way, the *Y* coordinate can be obtained.

To obtain the *X* (or *Y*) coordinate, one divider, three adders, six multiplexers, six flipflops (two flipflops are public), and 32 selectors are applied. After the processing of the RaM module, the *X* coordinate and *Y* coordinate are acquired with the done signal, RaM_Done_Sig, in the same clock cycle.

**• Image Coordinate Calculation Module**

When the RaM_Done_Sig, *X*, and *Y* coordinates are being detected, the RCM module starts to calculate the image coordinates (*Samp*, *Line*), i.e., column and row indexes. As shown in Equation (9), the equations give the relationship between the normalized coordinates (*X*, *Y*) and image coordinates (*Samp*, *Line*).

**Figure 9:**
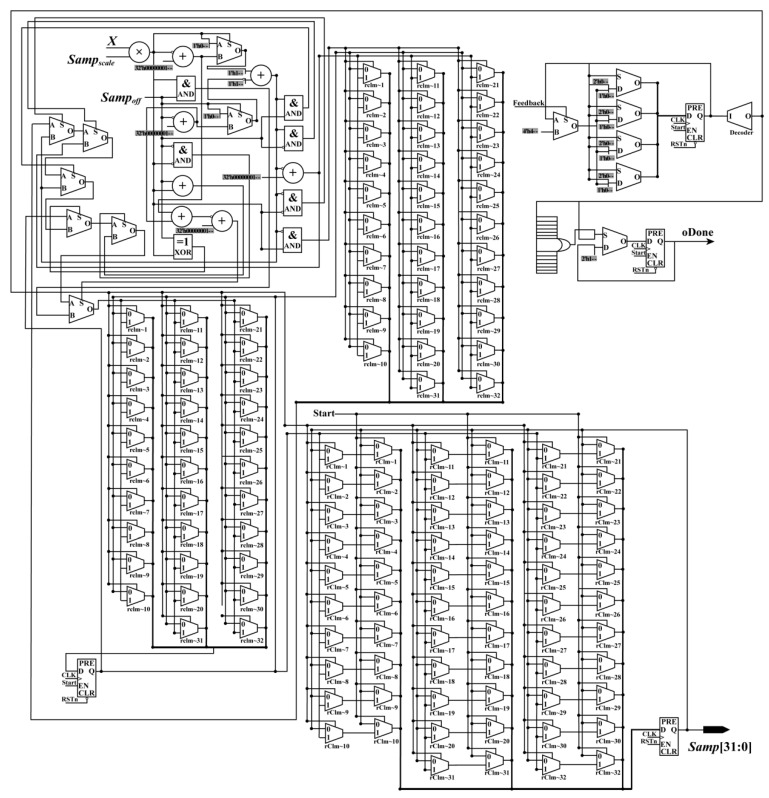
Schematic diagram used for calculating *Samp* coordinate.

As shown in Equation (9), the equations have a uniform form, which is helpful for implementation using an FPGA. To calculate the image coordinates (*Samp*, *Line*) in parallel, a parallel-computing hardware architecture is designed. Because the forms of the equations in Equation (9) are similar, only the schematic diagram used for calculating the *Samp* coordinate is given. As shown in Figure 9, there are one multiplier, four flipflops (two of them are shared when calculating *Line* coordinate), five selectors (MUX) shared when calculating *Line* coordinate, seven adders, and 136 multiplexers (MUX21).

After the processing of the RCM module, the image coordinates, that is, the column and row indexes (*Samp*, *Line*), and the done signal (RCM_Done_Sig) are obtained in the same clock cycle. Up to this point, the whole processing of the coordinate transformation is done. The obtained image coordinates are sent to the interpolation module to interpolate the grayscale.

#### 2.2.3. Interpolation Module

Because the obtained column and row indexes may not exist only at the center of a pixel, it is necessary to use the interpolation method to obtain the grayscale in the obtained column and row indexes. Considering the interpolation effect, the complexity of an interpolation algorithm, and the resources of an FPGA, the bilinear interpolation method is selected to implement the interpolation for grayscale. Mathematically, the bilinear interpolation algorithm can be expressed by the following equation:

(18) $$\begin{array}{ll} g(i+p,\;j+q)= & \left(1-p)(1-q)g(i,\;j)+(1-p)qg(i,\;j+1\right)\\ & +p(1-q)g(i+1,\;j)+pqg(i+1,\;j+1) \end{array}$$

where *i* and *j* are nonnegative integers; the intermediates *p* = |*i* − int(*i*)| and *q* = |*j* − int(*j*)| are within the range of (0, 1); and *g*(*i*, *j*) represents gray values.

**Figure 10:**
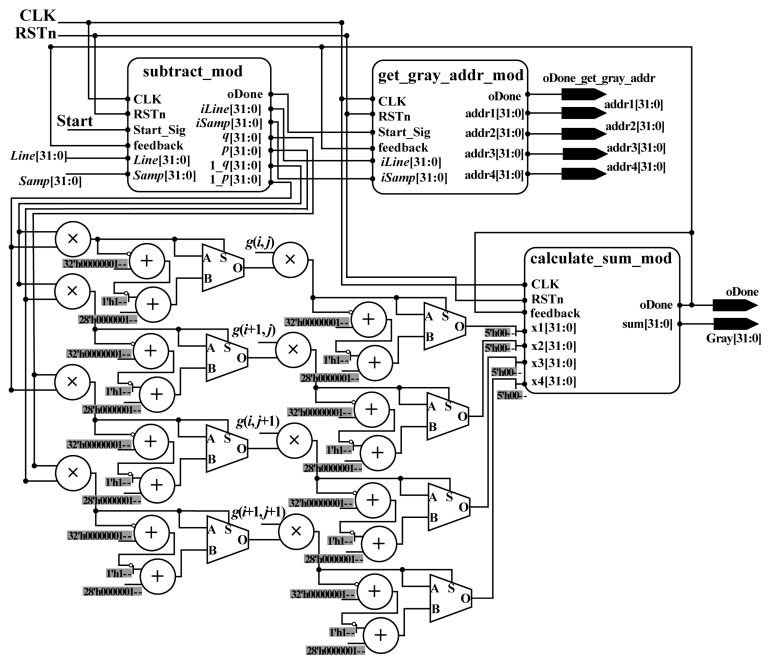
Schematic diagram of interpolation module.

To implement the bilinear interpolation algorithm in parallel using an FPGA chip, a parallel computation architecture was designed (see Figure 10). The designed hardware architecture contains four submodules/parts: (i) the subtract_mod, which is used to obtain the integer part (*iLine* and *iSamp*) and fractional part (*p* and *q*) of *Line* and *Samp* indexes, and to calculate the subtraction (1_*p* and 1_*q*) in Equation (18); (ii) the get_gray_addr_mod, which is applied to obtain the address of gray in RAM; (iii) the multiplication part, which is utilized to calculate the multiplications in Equation (18); and (iv) the calculate_sum_mod, which is used to compute the sum in Equation (18). After the processing of the calculate_sum_mod, the results of interpolation in (*Samp*, *Line*) are obtained. The details of subtract_mod, get_gray_addr_mod, and calculate_sum_mod are described as follows.

**• subtract_mod**

**Figure 11:**
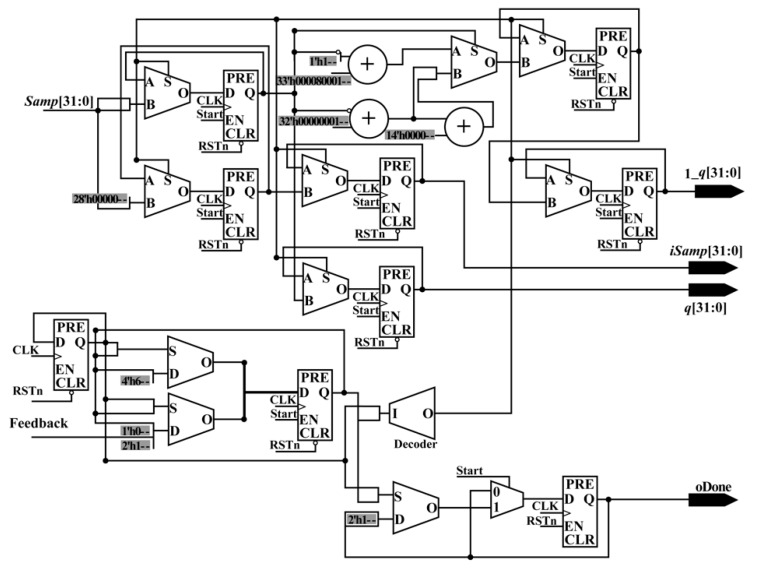
Schematic diagram of the subtract_mod module.

As shown in Equation (18), to perform the bilinear interpolation method, the gray values of four neighbors around the acquired column and row indexes are required. Thus, the acquired column and row indexes should be pre-processed to obtained the integer part and fractional part, which are used to calculate (1 − *q*) and (1 − *p*). To implement the function using an FPGA chip, a parallel-computing architecture is proposed, named subtract_mod. In subtract_mod, the methods used to acquire *iSamp*, *q* and 1*_q* are similar to those for obtaining *iLine*, *p* and 1*_p*, respectively. Thus, in this section, only the schematic diagram for obtaining *iSamp*, *q* and 1*_q* is given (see Figure 11).

As shown in Figure 11, to obtain *iSamp*, *q* and 1*_q*, three adders, seven multiplexers (MUX21), and nine flipflops (three of which are shared when *iLine*), *p* and 1*_p* are used. In addition, three MUXs are public. After the processing of the whole subtract_mod, *iSamp*, *q*, 1*_q*, *iLine*, *p*, and 1*_p* are acquired with the done signal in the same clock cycle. When obtaining these variables, *iSamp* and *iLine* are sent to the next submodule to retrieve the address of gray for four neighbors in RAM. Meanwhile, *q*, 1*_q*, *iLine*, *p*, and 1*_p* are sent to another part to perform multiplication.

**• get_gray_addr_mod**

**Figure 12:**
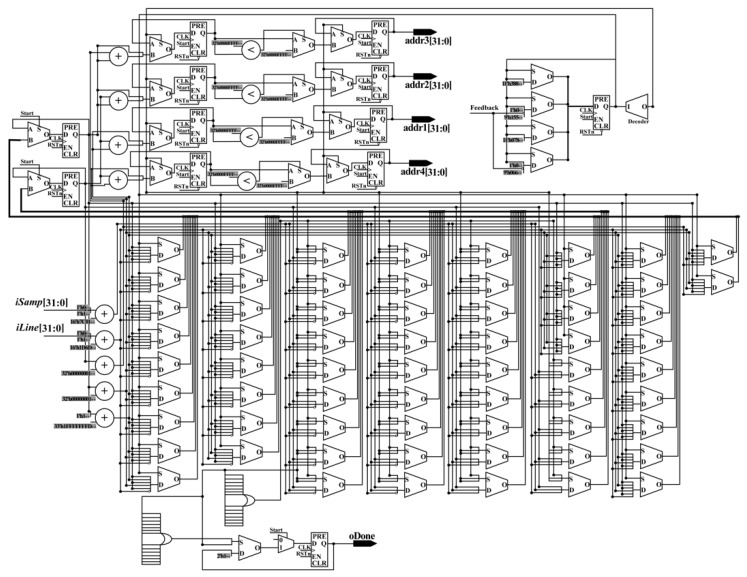
Schematic diagram of the get_gray_addr_mod module.

The grayscale of a pixel can be obtained according to the corresponding address. To obtain the gray values of four neighbors around the obtained column and row indexes in parallel, a parallel-computing hardware architecture is proposed (see Figure 12), called get_gray_addr_mod. In the get_gray_addr_mod, 3 LESS-THAN comparators, 9 adders, 10 MUX21, 12 flipflops, and 70 MUX are applied. After the processing of the get_gray_addr_mod, four addresses are obtained with the done signal in the same clock cycle. According to the obtained addresses, the gray values can be acquired from RAM. Then, they are sent to the multiplication part to perform the multiplication.

**• calculate_sum_mod**

As shown in Figure 10, after the multiplication process, four variables, *x*_1_, *x*_2_, *x*_3_, and *x*_4_, are obtained in the same clock cycle. To implement the addition for four variables, two levels of additions are needed. Each addition corresponds to an extended addition that has an architecture that is similar to Figure 7. The details can be found in Section 2.2.2 and Figure 7. After the processing of the calculate_sum_mod, the result of interpolation in (*Samp*, *Line*) can be obtained.

### 2.3. Integration On-Board System

An FPGA device can be integrated into the on-board system as a part of the system, because FPGA has advantages in size, weight, and power. After completing the proposed algorithm design using Verilog language, the designed algorithm can be programmed into the selected FPGA device.

## 3. Experiments

### 3.1. Software and Hardware Environment

**Figure 13:**
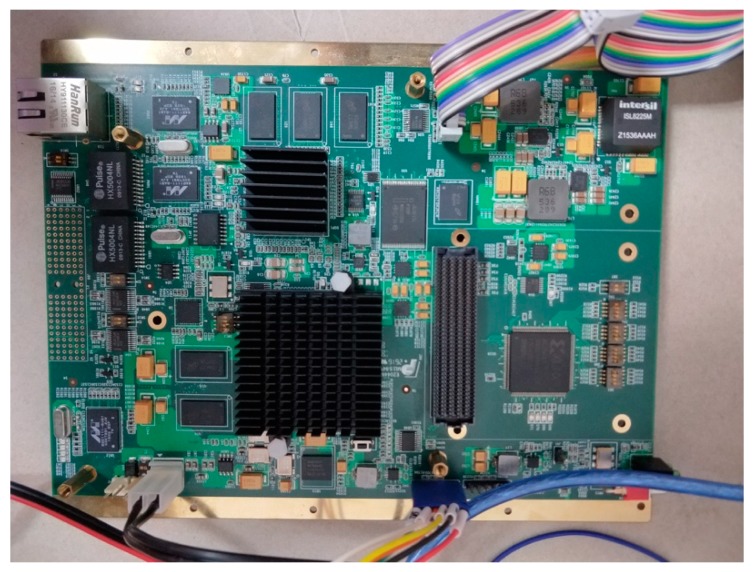
Photograph of the FPGA platform.

In this study, an Altera FPGA was used. The version of the FPGA is Kintex-7 XC7K325TFFG900-1 (see Figure 13), the design tool is Vivado 2016.4 (Xilinx, San Jose, CA, USA), and the simulation tool is ModelSim SE10.1d (Mentor, Santa Barbara, CA, USA). The PC uses a Windows 7 (64 bit) operating system, and has an Intel^®^ Core™ i7-4790 CPU @ 3.6 GHz processor with 8 GB RAM. To validate the proposed method, the orthorectification algorithm was also implemented using Matlab 2012a (MathWorks, 1 Apple Hill Drive, Natick, MA, USA).

### 3.2. Dataset

**Figure 14:**
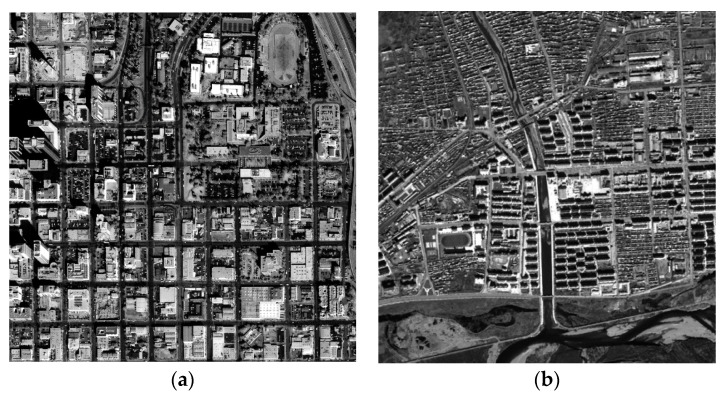
(**a**) Original IKONOS image; and (**b**) original SPOT6 image.

To validate the correction accuracy and processing speed of the proposed FPGA-based orthorectification method, two test datasets (as shown in Figure 14) were used in this study. The first study area is located in San Diego, CA, USA. The IKONOS-2 PAN image with the resolution of 1.0 m was collected on 7 February 2000. The wavelength range of IKONOS-2 PAN image is 450–900 nm. The second study area is located in Genhe, Inner Mongolia, China. The SPOT-6 PAN image with the resolution of 1.5 m was acquired on 29 September 2013. The wavelength range of SPOT-6 PAN image is 450–745 nm. The known parameters are listed in Table 2, Table 3 and Table 4.

According to the proposed method, input parameters should be transformed into fixed-point data. As shown in Table 2, Table 3 and Table 4, the values of parameters of two study areas are in different range. To ensure computation accuracy, all parameters are transformed into fixed-point of 32 bits using different scale factor, *τ*. The details are given in Table 5 and Table 6. In addition, the clock frequency is 100 MHz.

### 3.3. Results

**Figure 15:**
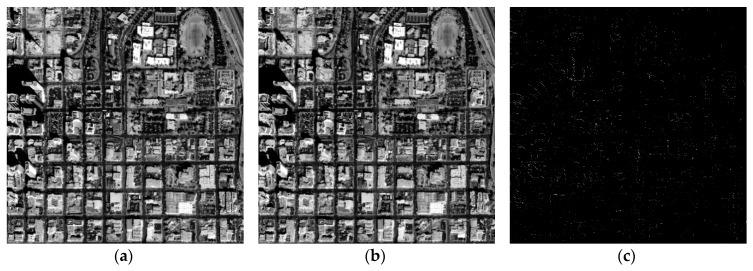
Orthoimages for the first study area: (**a**) by FPGA; and (**b**) by Matlab; and (**c**) the difference of image (**a**) and image (**b**).

**Figure 16:**
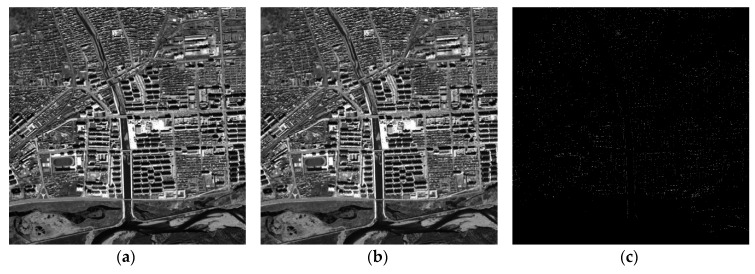
Orthoimages for the second study area: (**a**) by FPGA; and (**b**) by Matlab; and (**c**) the difference of image (**a**) and image (**b**).

As shown in Figure 15a and Figure 16a, after the processing of the proposed method, the orthorectified results (orthophoto) were obtained. To validate the accuracy and speed of the proposed rectification method, orthorectification for the same datasets was also implemented by applying the PC-based platform. On the PC-based platform, the proposed FP-RPC orthorectification was implemented using Matlab codes.

The orthorectification results obtained using the PC-based software are shown in Figure 15b and Figure 16b. The contrast-enhanced difference images for two study areas are shown in Figure 15c and Figure 16c, respectively. As shown in Figure 15c and Figure 16c, the contrast-enhanced difference images show the few discrepancies that are present. The orthorectification images obtained by FPGA and PC are not visually different by inspection. The numerical differences between FPGA and PC become apparent when observing the difference images shown in Figure 15c and Figure 16c. These pixel position differences are mainly caused by the used bit wide and scale factor of fixed-point data [1]. According to [1], the pixel position difference can be decreased with the increasing of bit wide and scale factor. Error analysis between the proposed method and the FP-RPC algorithm implemented on PC are provided in the next section.

## 4. Discussion

### 4.1. Error Analysis

To quantitatively evaluate the accuracy of the proposed orthorectification method, the root-mean-square error (RMSE) [40,41] was utilized. Mathematically, the RMSEs of the image coordinates along the vertical axis (Δ*I*) and horizontal axis (Δ*J*), and distance (Δ*S*) can be calculated using the following equations, respectively,

(19) $$\Delta I=\sqrt{\frac{\sum_{h=1}^{n}{\left({I^{\prime }}_{h}-I_{h}\right)}^{2}}{n-1}}\;\Delta J=\sqrt{\frac{\sum_{h=1}^{n}{\left({J^{\prime }}_{h}-J_{h}\right)}^{2}}{n-1}}$$

(20) $$\Delta S=\sqrt{\frac{\sum_{h=1}^{n}({\left({I^{\prime }}_{h}-I_{h}\right)}^{2}+{\left({J^{\prime }}_{h}-J_{h}\right)}^{2}}{n-1}}$$

where *I*′*_h_* and *J*′*_h_* are the image coordinates rectified by the proposed orthorectification method; *I_h_* and *J_h_* are the reference image coordinates; and *n* is the number of check points.

**Figure 17:**
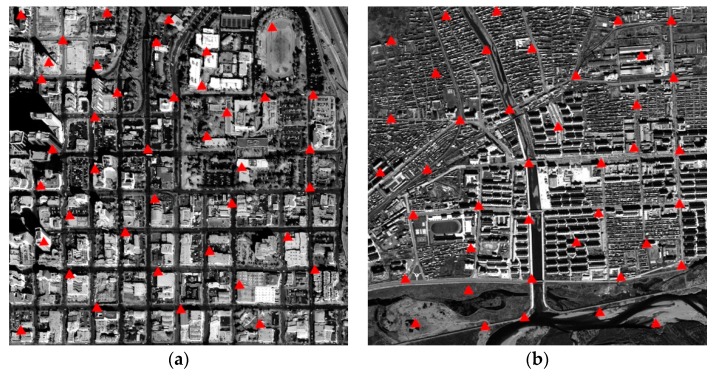
Check-point distribution in: (**a**) the first study area; and (**b**) the second study area.

**Figure 18:**
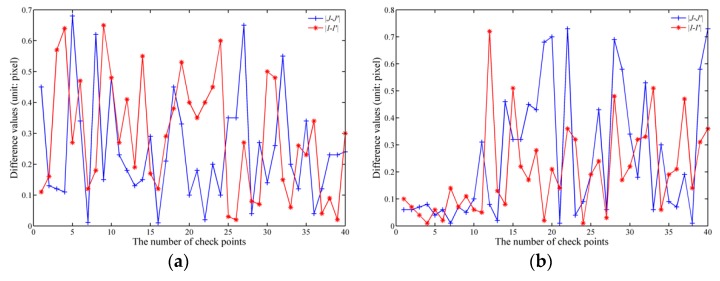
Different statistical analyses for the FPGA-based method and Matlab-based method for: (**a**) the first study area; and (**b**) the second study area.

To compute the RMSEs, 40 check points for each study area were selected randomly (as shown in Figure 17). As shown in Figure 18, the differences in the values of image coordinates for the Matlab-based and FPGA-based methods are given. Based on Equations (19) and (20), the RMSEs (Δ*I*, Δ*J*, and Δ*S*) are 0.35 pixels, 0.30 pixels, and 0.46 pixels, respectively, for the first study area; meanwhile, they are 0.27 pixels, 0.36 pixels, and 0.44 pixels, respectively, for the second study area. Moreover, other statistics were also calculated (as shown in Table 7).

According to the calculation results of Equations (19) and (20), the orthorectification results obtained using the proposed method are considered acceptable because the RMSEs are less than one pixel [42,43,44]. However, as shown in Figure 18, differences still exist in the image coordinates acquired by the FPGA-based and Matlab-based methods. These differences may be caused by the algorithms implemented by FPGA hardware, for example, the fixed-point computation, which propagate and accumulate.

### 4.2. Processing Speed Comparison

This section presents the processing time as the size of satellite image increases, and evaluates processing speed of the FPGA-based orthorectification method and the Matlab-based method.

**Figure 19:**
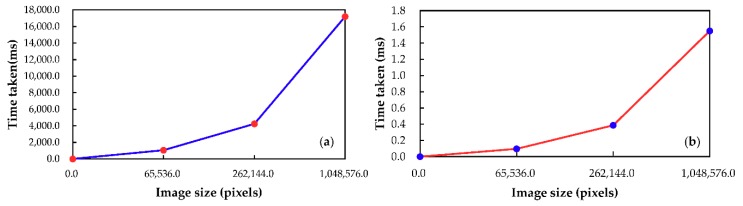
Image size vs. time taken to perform RPC orthorectification algorithm: (**a**) Matlab-based platform; and (**b**) FPGA-based platform.

The processing time have been recorded as an average of 10 runs of the RPC orthorectification algorithm for each image. The average processing time for difference size of image is presented in Table 8. A plot of the image size vs. processing time is shown in Figure 19. The speed-up of the method can be defined as the Matlab time taken divided by the time taken on the FPGA for the performance of the RPC algorithm [45]. In the image case considered, the maximum speed-up is acquired from a size of 1024 × 1024 pixels, where the speed-up is about 11,095.8709. From the results in Table 8, it can be demonstrated that the speed increases with the size of image.

The processing speed is one of the most importance indicators for evaluating on-board processing. To evaluate and compare the speed of the proposed FPGA-based orthorectification method and the Matlab-based method, the throughput, which is a normalized metric, is used, and represents the capacity in terms of the number of pixels processed per second. For the proposed method, the average throughput is approximately 675.67 Mpixels/s. However, for the Matlab-based method, the average throughput is approximately 61,677.49 pixels/s. This means that the proposed FPGA-based method has higher processing capacity than the Matlab-based method.

### 4.3. Resource Consumption

Besides the speed of processing, the utilization ratio of each type of resource is also a key indicator when assessing the quality of a method. As is well known, it can be determined whether a selected device meets the requirement of a design scheme by analyzing the utilization ratio of hardware resource. If the utilization ratio of a type of resource reaches 60–80%, the selected device satisfies the requirement of the design scheme.

Thus, after implementing the proposed method, some important resources, such as look-up tables (LUTs), registers, and total pins are analyzed. As shown in Table 9, the slice logics contain slice LUTs and slice registers. The utilization ratios of LUTs and registers are 44.42% and 5.59%, respectively. The utilization of input and output (IO) is 368, which is 73.60% of the total IOs.

In short, according to the above comprehensive utilization ratios for resources, it can be demonstrated that the resources of the selected FPGA can meet the design requirement of the proposed FPGA-based orthorectification method.

## 5. Conclusions

This paper proposes an orthorectification method, namely, the field-programmable gate array (FPGA)-based fixed-point (FP) rational polynomial coefficient (RPC) method (FPGA-based FP-RPC method) to perform the process of orthorectification on board spacecraft/satellite to accelerate the orthorectification processing speed for remotely sensed (RS) images. The proposed FPGA-based FP-RPC method contains three main submodules, Read_parameter_mod, Coordinate_transform_mod, and Interpolation_mod, based on the bilinear interpolation algorithm.

To validate the orthorectification accuracy, an orthophoto that was orthorectified by a PC-based platform (Matlab 2012a) was used as a reference. Two datasets, IKONOS and SPOT-6 images, were used to validate the proposed FPGA-based FP-RPC method. The root-mean-square error (RMSE), which is associated with the maximum, minimum, standard deviation (STD), and mean of row and column coordinates’ differences, was used. The experimental results show that the STD of the row and column coordinates’ differences are 0.19 pixels and 0.18 pixels, respectively, for the first study area, while they were 0.16 pixels and 0.24 pixels, respectively, for the second study area. The RMSE of the row coordinate (Δ*I*), column coordinate (Δ*J*) and the distance Δ*S* are 0.35 pixels, 0.30 pixels, and 0.46 pixels, respectively, for the first study area, while they are 0.27 pixels, 0.36 pixels, and 0.44 pixels, respectively, for the second study area. It can be concluded from these quantitative analyses that the proposed method can meet the demand of orthorectification in practice.

Moreover, a comparison of the processing speed was also performed for the proposed FPGA-based FP-RPC method and PC-based RPC methods. The throughput of the FPGA-based FP-RPC method and PC-based RPC method are 675.67 Mpixels/s and 61,070.24 pixels/s, respectively. Therefore, it can be shown that the processing speed of the FPGA-based FP-RPC method is faster (by approximately 11,000 times) than the processing speed of the Matlab-based RPC method. In terms of the resource consumptions, it can be found that the utilization ratios of ALUTs, registers, and IO are 44.42%, 5.59%, and 73.60%, respectively.

## Figures and Tables

**Table 1:** Scale factors and integer variables.

Variable Name	Scale Factor	Integer Variable Name
*a*′*_i_*, *b*′*_i_*, *c*′*_i_*, and *d*′*_i_* (*i* = 1 to 20)	*τ*_1_	*a_i_*, *b_i_*, *c_i_*, and *d_i_* (*i* = 1 to 20)
*Lon*′, *Lat*′, *Hei*′	*τ*_2_	*Lon*, *Lat*, *Hei*
*Lat*′*_off_*, *Lat*′*_scale_*, *Lon*′*_off_*, *Lon*′*_scale_*, *H*′*_off_*, *H*′*_scale_*	*τ*_3_	*Lat_off_*, *Lat_scale_*, *Lon_off_*, *Lon_scale_*, *H_off_*, *H_scale_*
*Line*′*_off_*, *Line*′*_scale_*, *Samp*′*_off_*, *Samp*′*_scale_*	*τ*_3_	*Line_off_*, *Line_scale_*, *Samp_off_*, *Samp_scale_*

**Table 2:** Normalized parameters.

#	First Area	Second Area
*Line_off_* (pixels)	1135	24,874.5
*Samp_off_* (pixels)	2548	17,962.5
*Lat_off_* (degrees)	32.718700	50.737358
*Lon_off_* (degrees)	−117.13340	121.44648
*H_off_* (meters)	36.000	500
*Line_scale_* (pixels)	1829	24,874.5
*Samp_scale_* (pixels)	6570	17,962.5
*Lat_scale_* (degrees)	0.01710000	0.41058849
*Lon_scale_* (degrees)	0.07090000	0.45794952
*H_scale_* (meters)	223	500

**Table 3:** Rational function polynomial coefficients of the first study area.

*#*	Values	*#*	Values	*#*	Values	*#*	Values
***a*_1_**	−7.52883250 × 10^−4^	***b*_1_**	1	***c*_1_**	−9.23491680 × 10^−4^	***d*_1_**	1
***a*_2_**	4.60115225 × 10^−3^	***b*_2_**	−1.68736561 × 10^−3^	***c*_2_**	1.01134804	***d*_2_**	−1.68736561 × 10^−3^
***a*_3_**	−1.03642070	***b*_3_**	1.88384395 × 10^−3^	***c*_3_**	3.57115249 × 10^−4^	***d*_3_**	1.88384395 × 10^−3^
***a*_4_**	−3.93943040 × 10^−2^	***b*_4_**	−6.55340329 × 10^−4^	***c*_4_**	−1.17541741 × 10^−2^	***d*_4_**	−6.55340329 × 10^−4^
***a*_5_**	1.75874570 × 10^−3^	***b*_5_**	2.11928788 × 10^−7^	***c*_5_**	1.71162003E × 10^−3^	***d*_5_**	2.11928788 × 10^−7^
***a*_6_**	2.25762210 × 10^−4^	***b*_6_**	−2.15886792 × 10^−7^	***c*_6_**	−2.77384658 × 10^−4^	***d*_6_**	−2.15886792 × 10^−7^
***a*_7_**	6.47497342 × 10^−4^	***b*_7_**	6.60194370 × 10^−8^	***c*_7_**	−4.67286564 × 10^−5^	***d*_7_**	6.60194370 × 10^−8^
***a*_8_**	−1.22344418 × 10^−3^	***b*_8_**	1.29969058 × 10^−6^	***c*_8_**	−1.70712175 × 10^−3^	***d*_8_**	1.29969058 × 10^−6^
***a*_9_**	−1.95386510 × 10^−3^	***b*_9_**	−6.96485750 × 10^−7^	***c*_9_**	7.61699396 × 10^−7^	***d*_9_**	−6.96485750 × 10^−7^
***a*_10_**	2.70799645 × 10^−5^	***b*_10_**	3.41030606 × 10^−7^	***c*_10_**	2.98181047 × 10^−6^	***d*_10_**	3.41030606 × 10^−7^
***a*_11_**	5.10672113 × 10^−7^	***b*_11_**	5.17265975 × 10^−10^	***c*_11_**	8.68077245 × 10^−7^	***d*_11_**	5.17265975 × 10^−10^
***a*_12_**	2.05965613 × 10^−6^	***b*_12_**	2.71171743 × 10^−10^	***c*_12_**	1.42413537 × 10^−6^	***d*_12_**	2.71171743 × 10^−10^
***a*_13_**	−2.18726634 × 10^−7^	***b*_13_**	−1.47633205 × 10^−10^	***c*_13_**	−1.11312088 × 10^−6^	***d*_13_**	−1.47633205 × 10^−10^
***a*_14_**	5.40855097 × 10^−8^	***b*_14_**	3.59570414 × 10^−10^	***c*_14_**	2.35665930 × 10^−7^	***d*_14_**	3.59570414 × 10^−10^
***a*_15_**	−3.96996732 × 10^−6^	***b*_15_**	2.28588675 × 10^−10^	***c*_15_**	5.40107978 × 10^−7^	***d*_15_**	2.28588675 × 10^−10^
***a*_16_**	7.19308892 × 10^−7^	***b*_16_**	−1.11864088 × 10^−10^	***c*_16_**	−1.10872161 × 10^−10^	***d*_16_**	−1.11864088 × 10^−10^
***a*_17_**	−3.89372910 × 10^−7^	***b*_17_**	−1.37823694 × 10^−10^	***c*_17_**	−4.86264967 × 10^−10^	***d*_17_**	−1.37823694 × 10^−10^
***a*_18_**	−4.18443985 × 10^−6^	***b*_18_**	−3.32951199 × 10^−9^	***c*_18_**	−8.43531861 × 10^−7^	***d*_18_**	−3.32951199 × 10^−9^
***a*_19_**	4.50802285 × 10^−8^	***b*_19_**	6.33689691 × 10^−10^	***c*_19_**	−3.64346611 × 10^−8^	***d*_19_**	6.33689691 × 10^−10^
***a*_20_**	−1.57227534 × 10^−8^	***b*_20_**	−5.49482473 × 10^−11^	***c*_20_**	−2.38643375 × 10^−9^	***d*_20_**	−5.49482473 × 10^−11^

**Table 4:** Rational function polynomial coefficients of the second study area.

*#*	Values	*#*	Values	*#*	Values	*#*	Values
***a*_1_**	0.00207581	***b*_1_**	1	***c*_1_**	−0.01727220	***d*_1_**	1
***a*_2_**	0.05939323	***b*_2_**	5.06562471 × 10^−9^	***c*_2_**	1.01955596	***d*_2_**	−2.43637767 × 10^−6^
***a*_3_**	−1.06139835	***b*_3_**	−2.23329117 × 10^−9^	***c*_3_**	0.00149223	***d*_3_**	1.76928700 × 10^−6^
***a*_4_**	0.00300505	***b*_4_**	−2.21235982 × 10^−11^	***c*_4_**	−0.00498582	***d*_4_**	−1.29701506 × 10^−7^
***a*_5_**	9.99447200 × 10^−5^	***b*_5_**	−3.85166222 × 10^−8^	***c*_5_**	−0.01544990	***d*_5_**	−4.50015117 × 10^−5^
***a*_6_**	4.48210655 × 10^−6^	***b*_6_**	7.22875706 × 10^−11^	***c*_6_**	0.00070933	***d*_6_**	1.03746933 × 10^−6^
***a*_7_**	−0.00011601	***b*_7_**	1.96276656 × 10^−9^	***c*_7_**	−0.00021711	***d*_7_**	−2.16896140 × 10^−6^
***a*_8_**	−0.00285285	***b*_8_**	1.03082157 × 10^−8^	***c*_8_**	0.01454522	***d*_8_**	2.27253718 × 10^−5^
***a*_9_**	−0.00025849	***b*_9_**	4.58273834 × 10^−8^	***c*_9_**	0.00146642	***d*_9_**	2.71848287 × 10^−5^
***a*_10_**	8.66439267 × 10^−8^	***b*_10_**	4.12786890 × 10^−12^	***c*_10_**	−2.61417544 × 10^−6^	***d*_10_**	−1.20465997 × 10^−7^
***a*_11_**	1.94846265 × 10^−7^	***b*_11_**	−8.77450090 × 10^−11^	***c*_11_**	−2.38142758 × 10^−5^	***d*_11_**	−8.45078827 × 10^−9^
***a*_12_**	7.22766464 × 10^−7^	***b*_12_**	−1.25925035 × 10^−9^	***c*_12_**	4.37422992 × 10^−5^	***d*_12_**	−2.25373082 × 10^−9^
***a*_13_**	−2.45655192 × 10^−5^	***b*_13_**	−1.41399102 × 10^−9^	***c*_13_**	−0.00012373	***d*_13_**	1.66156835 × 10^−7^
***a*_14_**	−2.15776820 × 10^−10^	***b*_14_**	1.09626013 × 10^−12^	***c*_14_**	3.1682470454 × 10^−7^	***d*_14_**	−2.87815787 × 10^−10^
***a*_15_**	3.53191253 × 10^−5^	***b*_15_**	1.82192638 × 10^−9^	***c*_15_**	0.00022374	***d*_15_**	−1.35493121 × 10^−7^
***a*_16_**	3.58935305 × 10^−5^	***b*_16_**	−8.74976255 × 10^−10^	***c*_16_**	1.88815906 × 10^−5^	***d*_16_**	−2.31068042 × 10^−8^
***a*_17_**	−3.00220333 × 10^−9^	***b*_17_**	2.05074275 × 10^−13^	***c*_17_**	−1.37072926 × 10^−7^	***d*_17_**	5.66675066 × 10^−10^
***a*_18_**	−3.32028434 × 10^−7^	***b*_18_**	8.02116204 × 10^−11^	***c*_18_**	1.95362054 × 10^−5^	***d*_18_**	−2.37161908 × 10^−9^
***a*_19_**	−1.44250161 × 10^−7^	***b*_19_**	−1.15845372 × 10^−10^	***c*_19_**	2.68897003 × 10^−6^	***d*_19_**	6.41890372 × 10^−9^
***a*_20_**	1.88636696 × 10^−12^	***b*_20_**	5.66352056 × 10^−16^	***c*_20_**	−1.15446432 × 10^−9^	***d*_20_**	−7.23372539 × 10^−12^

**Table 5:** Scale factors for parameters of First Area.

	*τ*	Range	Accuracy
*Lat*’, *Lon*’, *Hei*’	23	(−256, 255.999999881)	0.000000119
*Lat*′*_off_*, *Lat*′*_scale_*, *Lon*′*_off_*, *Lon*′*_scale_*	23	(−256, 255.999999881)	0.000000119
*H*′*_off_*, *H*′*_scale_*	23	(−256, 255.999999881)	0.000000119
*Line*′*_off_*, *Line*′*_scale_*, *Samp*′*_off_*, *Samp*′*_scale_*	18	(−8192, 8191.999996185)	0.000003815
*a*′*_i_*, *b*′*_i_*, *c*′*_i_*, and *d*′*_i_* (*i* = 1 to 20)	30	(−2, 1.999999999)	0.000000001

**Table 6:** Scale factors for parameters of Second Area.

	*τ*	Range	Accuracy
*Lat*′, *Lon*′, *Hei*′	23	(−256, 255.999999881)	0.000000119
*Lat*′*_off_*, *Lat*′*_scale_*, *Lon*′*_off_*, *Lon*′*_scale_*	23	(−256, 255.999999881)	0.000000119
*H*′*_off_*, *H*′*_scale_*	21	(−1024, 1023.999999523)	0.000000477
*Line*′*_off_*, *Line*′*_scale_*, *Samp*′*_off_*, *Samp*′*_scale_*	16	(−32,768, 32,767.999984741)	0.000015259
*a*′*_i_*, *b*′*_i_*, *c*′*_i_*, and *d*′*_i_* (*i* = 1 to 20)	30	(−2, 1.999999999)	0.000000001

**Table 7:** Statistical analysis for the different image coordinates obtained by Matlab and FPGA (unit: pixel).

Study Area No.		Maximum	Minimum	Mean	STD
1st	|*I*′-*I*|	0.65	0.02	0.29	0.19
	|*J*′-*J*|	0.68	0.01	0.25	0.18
2nd	|*I*′-*I*|	0.72	0.01	0.20	0.16
	|*J*′-*J*|	0.73	0.01	0.26	0.24

**Table 8:** Average processing time for RPC orthorectification implementation on Matlab and FPGA.

No.	Image Size (Pixels)	Matlab Time (s)	FPGA Time (ms)	Speed-Up
1	256 × 256	1.0515	0.09686	10,855.8745
2	512 × 512	4.2461	0.3875	11,008.8151
3	1024 × 1024	17.1986	1.5500	11,095.8709

**Table 9:** Utilization ratio of resources for the proposed FPGA-based orthorectification.

#		Utilization	Available	Utilization Ratio (%)
Slice logic	Slice LUTs	90,634	203,800	44.42
	Slice registers	22,798	407,600	5.59
IO		368	500	73.60
